# *Limosilactobacillus reuteri* HM108 alleviates obesity in rats fed a high-fat diet by modulating the gut microbiota, metabolites, and inhibiting the JAK-STAT signalling pathway

**DOI:** 10.3389/fnut.2025.1597334

**Published:** 2025-06-25

**Authors:** Mi Tang, Xianping Li, Jiahui Ren, Chunyu Yao, Lu Liu, Xiaojing Li, Xueping Yuan, Junying Zhao, Bin Liu, Weicang Qiao, Lijun Chen

**Affiliations:** ^1^Key Laboratory of Dairy Science, Ministry of Education, Food Science College, Northeast Agricultural University, Harbin, China; ^2^National Engineering Research Center of Dairy Health for Maternal and Child, Beijing Sanyuan Foods Co., Ltd., Beijing, China; ^3^Beijing Engineering Research Center of Dairy, Beijing Technical Innovation Center of Human Milk Research, Beijing Sanyuan Foods Co., Ltd., Beijing, China; ^4^School of Bioengineering, Dalian Polytechnic University, Dalian, China

**Keywords:** *Limosilactobacillus reuteri*, obesity, high-fat diet, inflammation, gut microbiota, untargeted metabolomics, transcriptomics

## Abstract

**Introduction:**

Obesity is a globally prevalent metabolic disease, and high-calorie diets are major contributors to its development. Probiotic interventions can modulate the gut flora and alleviate systemic and low-grade inflammation, making them potential in-terventions for alleviating metabolic syndrome.

**Methods:**

This study explored the beneficial effects of the *Limosilactobacillus reuteri* HM108 strain derived from breast milk on obesity in high-fat diet-induced rats using a multi-gradient concentration in-tervention. Serum biochemical markers and inflammatory mediators were determined using enzyme-linked immunosorbent assay after 6-week intervention. Gut microbiota was assessed using 16S rRNA sequencing. The levels of short-chain fatty acid were detected using gas chromatography–mass spectrometry, fecal metabolites were ana-lysed using untargeted metabolomics, and the liver tissue was subjected to tran-scriptomics analysis.

**Results and discussion:**

The findings indicated that *L. reuteri* HM108 mitigated obesity, reduced blood lipids levels and immune factors, as well as altered the gut mi-crobiota composition, including reducing the Firmicutes/Bacteroidetes ratio. *L. reuteri* HM108 also inhibited the JAK-STAT signalling pathway. *L. reuteri* HM108 alleviates obesity caused by a high-fat diet in rats, offering a theoretical foun-dation and practical insights for utilizing this strain in obesity management.

## 1 Introduction

Obesity remains a serious global public health concern, linked to heightened risks of chronic conditions such as type 2 diabetes, hyperlipidaemia, cardiovascular disease, stroke, osteoarthritis, and certain cancers (1). The fundamental cause of obesity is an imbalance between energy intake and expenditure (2). To address obesity and its complications, numerous strategies have been devised, including exercise, surgery, and pharmacological interventions. However, dietary interventions remain the most common approach because of their affordability and low potential for adverse effects (3). Obesity in animal models induced by a chronic high-fat diet (HFD) has been extensively used to mimic human obesity (4). Consequently, various studies have investigated obesity risk factors and interventions, focusing on dietary strategies, identifying effective ingredients for obesity management, and exploring the underlying mechanisms.

Probiotics are increasingly recognised as a therapeutic strategy for obesity management (5), particularly strains of *Lactobacillus* and *Bifidobacterium* (6). Obese individuals exhibit distinct gut microbiome structures and functions compared to healthy individuals, with an increased Firmicutes/Bacteroidetes (F/B) ratio (7). This dysbiosis makes microbiome regulation a potential therapeutic target for obesity (8). Probiotic intake helps restore gut microbiota balance, modulate intestinal dysbiosis, and alleviate obesity-related symptoms (9). In addition, the gut microbiota plays a crucial role in nutrient acquisition and energy homeostasis (10). Certain genera within the Firmicutes and Bacteroidetes phyla ferment dietary fibre, producing short-chain fatty acids (SCFAs), such as butyrate, propionate, and acetate (11). These SCFAs contribute to host metabolism, immune modulation, and gut barrier integrity through receptor-mediated pathways (12), thereby exerting beneficial effects in obesity mitigation (13). Probiotics also influence weight regulation by modulating energy balance, enhancing satiety, strengthening the intestinal barrier, and impacting bile acid metabolism (14).

*Limosilactobacillus reuteri*, a versatile probiotic, colonises various host environments, including the human gut, skin, and breast milk (15). *L. reuteri* may counteract hyperlipidaemia and obesity by improving cholesterol and bile acid metabolism while reinforcing the intestinal barrier (16). However, probiotic effects vary among strains, even within the same species (17). For instance, *L. reuteri* ATCC PTA 4659 has been shown to reduce body weight in murine models, whereas strain L6798 induces weight gain (18). Further research is necessary to elucidate the specific mechanisms by which *L. reuteri* influences weight regulation.

In the current study, *L. reuteri* HM108, isolated from breast milk, demonstrated strong acid and bile salt tolerance along with hypoglycaemic capacity. This research used an HFD-induced obesity model in male, specific pathogen-free (SPF) Sprague–Dawley (SD) rats. By detecting blood lipid levels, histopathological changes, and inflammatory factors, the effects of this strain on HFD-induced inflammatory responses, intestinal microbiota composition, intestinal metabolites, and mRNA expression in liver tissues of model rats were evaluated. The findings offer fresh perspectives on the role of *L. reuteri* in obesity management and establish a theoretical foundation for developing functional foods. These insights pave the way for innovative approaches in dietary interventions aimed at improving gut health and metabolic conditions.

## 2 Materials and methods

### 2.1 Cultivation of *Limosilactobacillus reuteri* HM108

*Limosilactobacillus reuteri* HM108 is derived from healthy breast milk and is presently conserved at the China General Microbiological Culture Collection Center (CGMCC No. 26166). *Limosilactobacillus reuteri* HM108 was grown in de Man–Rogosa–Sharpe broth (Hopebio Company, Qingdao, China) at 37°C for 12 h and passaged twice to ensure viability and promote optimal growth. After being rinsed twice with sterile phosphate-buffered saline (PBS), the bacteria were adjusted to the target concentration for gavage in rats.

### 2.2 HFD-induced obesity rat model and probiotic intervention

**Figure 1:**
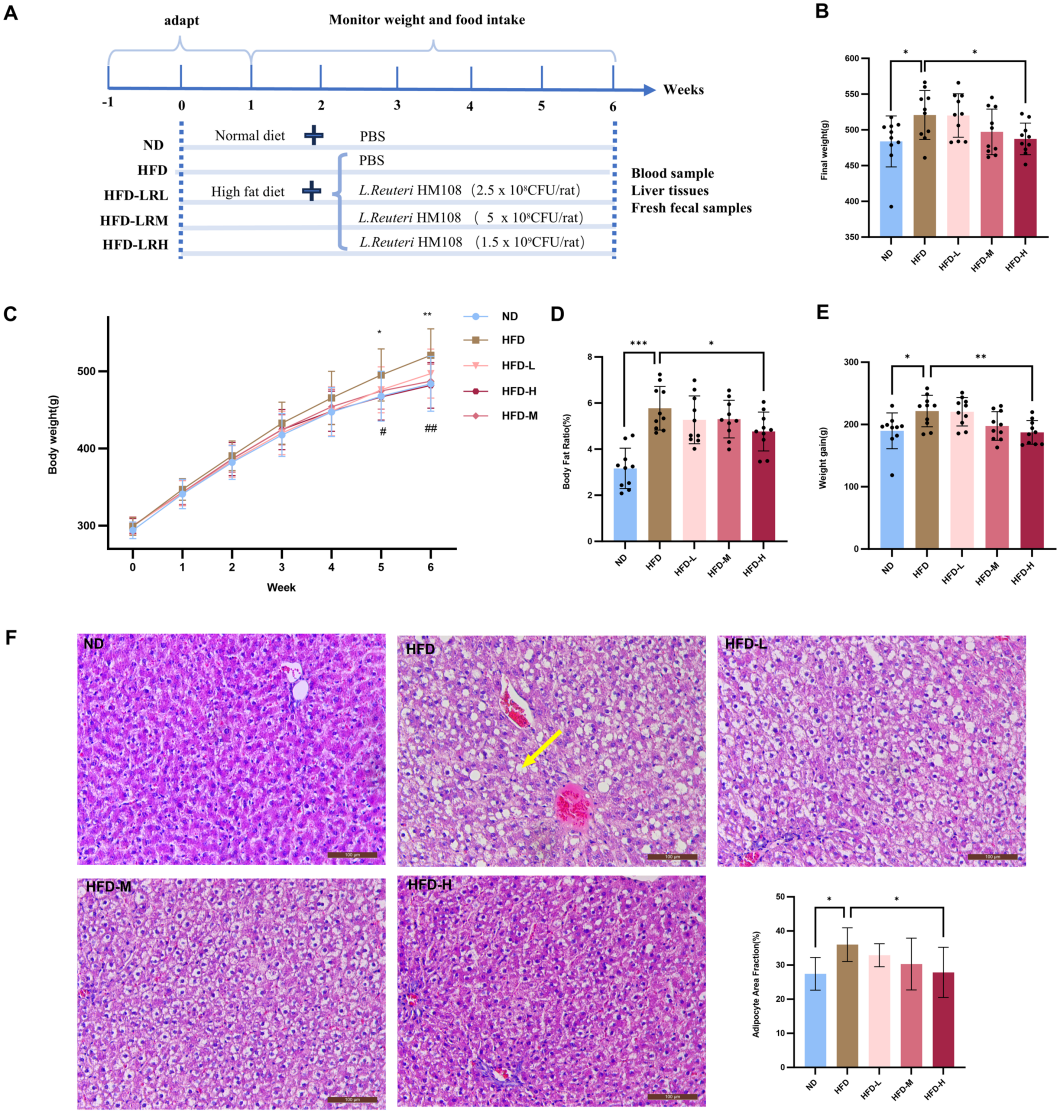
*L. reuteri* HM108 ameliorates HFD-induced weight gain and fat accumulation in rats. **(A)** Experimental design process. **(B)** Final body weight. **(C)** Body weight changes during the experiment. **(D)** Body fat ratio. **(E)** Weight gain from initial to final period. **(F)** H&E staining of liver adipose tissue and adipocyte area fraction using Image-Pro Plus 6.0 to estimate adipocyte size percentage (scale bar, 100 μm). Data are presented as mean ± SEM. Line graphs were analysed using two-way ANOVA, and histograms were analysed using unpaired *t*-tests. #Significant difference between HFD and HFD-M groups; * significant difference between HFD and HFD-H groups, * or #, *p* < 0.05, ** or ##, *p* < 0.01, ****p < 0.0001. ND, normal diet; HFD, high-fat diet; HFD-L, high-fat + low-dose (2.5 × 10^8^ CFU/rat); HFD-M, high-fat + medium-dose (5 × 10^8^ CFU/rat); HFD-H, high-fat + high-dose (1.5 × 10^9^ CFU/rat).

All animal experiments were conducted in line with the Guidelines for the Care and Use of Laboratory Animals of Beijing Union University and received approval from the Animal Ethics Committee of Beijing Union University (JCZX11-2306-2, Beijing, China). Male SD rats aged 6 weeks of SPF grade were sourced from Vital River Laboratory Animal Technology Co., Ltd. (Beijing, China). A detailed outline of the experimental protocol is presented in Figure 1A.

During the first week of acclimatisation, the animals were maintained at a constant temperature of 22 ± 2°C and a humidity level of 55 ± 10%. The animals were maintained on a normal diet (ND) under a 12-h light/dark regimen. Following this initial phase, 50 rats weighing 200 ± 20 g were divided randomly into five separate groups (*n* = 10): (1) ND; (2) HFD; (3) high-fat + low-dose (HFD-L, 2.5 × 10^8^ CFU/rat); (4) high-fat + medium-dose (HFD-M, 5 × 10^8^ CFU/rat); (5) high-fat + high-dose (HFD-H, 1.5 × 10^9^ CFU/rat), and three intervention groups supplemented with different concentrations of *L. reuteri* HM108 in their diet simultaneously. The HFD consisted of 15% sucrose and 15% lard. Over the course of the 6-w experiment, the ND and HFD groups of rats received a daily dose of 2 mL of pure PBS solution via gavage. The remaining groups were administered 2 mL of freshly prepared PBS containing the specified concentrations of *L. reuteri* HM108.

Following gavage, faecal samples were collected following a 12-h fasting period. Blood was extracted via cardiac puncture under sodium pentobarbital-induced anaesthesia. After resting for 1 h at 25°C, the serum was separated by centrifugation. The rats were then dissected to collect liver tissue, which was promptly flash-frozen in liquid nitrogen and preserved at −80°C for future analysis.

### 2.3 Determination of biochemical and inflammatory factors

Enzyme-linked immunosorbent assay (ELISA) kits (Nanjing Jiancheng Institute of Biological Engineering, Nanjing, China) were used to measure various biomarkers, including blood lipid contents—total cholesterol (TC), triglycerides (TG), high-density lipoprotein cholesterol (HDL-C), and low-density lipoprotein cholesterol (LDL-C)—as well as blood glucose, lipopolysaccharide (LPS), tumour necrosis factor-alpha (TNF-α), interleukin-1β (IL-1β), IL-10, and IL-6.

### 2.4 Histopathological analysis

Liver tissues were randomly selected, fixed in 10% formalin for 24 h, dehydrated, embedded in paraffin, sectioned into 5-μm slices, and stained with haematoxylin and eosin (H&E). Histological observations were performed using a light microscope, and the adipocyte area fraction was quantified (relative quantification) using Image-Pro Plus 6.0 (Media Cybernetics, Rockville, MD, United States).

### 2.5 16S rRNA measurement of intestinal microorganisms

The fresh, collected faeces were stored at −80°C. Gut microorganisms were measured and analysed by Shanghai Applied Protein Technology Co., Ltd. Briefly, the 16S rRNA V3–V4 region was amplified using primers 341F (CCTAYGGGRBGCASCAG) and 806R (TAATCTATGGGGNNCATAAGG). The Illumina Novaseq 6000 was used to conduct pyrosequencing of the polymerase chain reaction, followed by a rigorous quality assessment of the raw data, and merged paired-end reads.

The QIIME 2 package in R was used to select representative reads of each Amplicon Sequence Variant and perform alpha and beta diversity index analysis and visualisation. Finally, linear discriminant analysis effect size (LEfSe) was used to assess the importance of variations in species abundance and identify the different microbial biomarkers. The Kyoto Encyclopaedia of Genes and Genomes (KEGG) was used to predict the microflora metabolic function.

### 2.6 Gas chromatography-mass spectrometry analysis of SCFAs

The freshly-collected faeces were stored at −80°C, and SCFAs were measured and analysed by Shanghai Applied Protein Technology Co., Ltd. Briefly, the faeces were re-suspended in 50 μL of 20% phosphoric acid solution, with 4-methylpentanoic acid added as an internal standard. The mixtures were thoroughly agitated and then centrifuged, and the supernatant was carefully transferred into the gas chromatography-mass spectrometry (GC-MS) equipment (GC 7890 B, 5977 B, Agilent Technologies Co. Ltd., CA, United States) to determine SCFAs concentrations.

The samples were separated using a GC system equipped with an Agilent DB-FFAP capillary column (30 m × 250 μm, 0.25 μm). A 1-μL injection was performed using a 10:1 split ratio, with the column temperature initially set at 90°C. The temperature was increased to 160°C at a rate of 10°C/min, then to 240°C at 40°C/min, and held steady for 5 min. Helium was used as the carrier gas at a flow rate of 1.0 mL/min. MS followed, with inlet, ion source, and transmission line temperatures set at 250°C, 230°C, and 250°C, respectively, and a quadrupole temperature of 150°C. The electron ionisation source operated at 70 eV electron energy.

### 2.7 Untargeted faecal metabolism

The freshly collected faeces were stored at −80°C, and untargeted metabolomics were measured and analysed by Shanghai Applied Protein Technology Co., Ltd. Briefly, faecal samples were extracted with a cold methanol-acetonitrile-water (2:2:1, v/v), followed by vortexing and sonication. Finally, the supernatant was vacuum-dried, reconstituted in acetonitrile-water (1:1 v/v), centrifuged, and analysed using ultra-high-performance liquid chromatography coupled with a quadrupole time-of-flight system (UHPLC-QTOF) (Agilent 1290 Infinity/AB Sciex Triple TOF 6600).

The mobile phase consisted of two components: A (25 mM ammonium acetate/hydroxide in water) and B (acetonitrile). The gradient ran from 95% B (0.5 min) to 65% (6.5 min), then to 40% (1 min), held (1 min), returned to 95% (0.1 min), and re-equilibrated (3 min). Electrospray ionisation settings: ion source gas (Gas), curtain gas = 30, ion spray voltage = ±5,500 V. Primary *m*/*z* detection: 60–1,000 Da; secondary: 25–1,000 Da; collision energy = 35 ± 15 eV.

After sum-normalisation, the datasets were analysed using the ropls package in R and assessed using principal component analysis (PCA). The *t*-test was used to identify statistically significant differences between two independent sample groups. Significant differential metabolites were selected for KEGG pathway enrichment analysis.^1^

### 2.8 Liver tissue transcriptome analysis

The collected liver tissue samples were maintained at −80°C, and transcriptome was analysed by Shanghai Applied Protein Technology Co., Ltd. Briefly, liver RNA was quantified (A260/A280) using a Nanodrop ND-2000 system (Thermo Fisher Scientific, MA, United States). Sequencing was conducted using an Illumina Novaseq 6000. Using the HISAT2 package (version 2.2.1) in orientation mode, clean reads were individually aligned to the reference genome to acquire the mapped reads. The DESeq2 package (version 1.47.1) was analysed for differential expression genes (|log_2_ FC| >1 and *p*_adj_ <0.05). Finally, Gene Set Enrichment Analysis (GSEA) was conducted by calculating the enrichment scores, assessing their significance levels, and correcting for multiple hypothesis testing.

### 2.9 Statistical analysis

GraphPad Prism 10.1.2 was used for statistical analyses. A two-way analysis of variance followed by Dunnett’s test was used to analyse body weight. One-way analysis of variance (ANOVA) followed by Tukey’s test was used to analyse the differences in blood glucose and lipid content, inflammatory factors, proportion of SCFAs, and F/B ratio. Correlations between gut microbes and body fat ratio, blood lipid content, inflammatory factors, and differential metabolites were evaluated using Spearman’s correlation, with significance at *p* < 0.05.

## 3 Results

### 3.1 *Limosilactobacillus reuteri* HM108 mitigates weight gain in HFD rats

HFD feeding for 6 w substantially increased the total intake, total calories, weight gain, total body weight, body fat weight, body fat ratio, and feed intake of the rats (*p* < 0.05). In intervention groups with different doses of probiotics, there was no significant effect on the feed intake of rats, but the three parameters of body weight (*p* < 0.01), body fat weight, and body fat ratio (*p* < 0.05) showed dose-dependent improvements, indicating that supplementation with *L. reuteri* HM108 can significantly reduce high-fat diet induced weight gain and decrease body fat weight and body fat ratio (Figures 1B–E). Other indices were reduced to varying degrees, although not statistically (Supplementary Figures 1A–E).

Prolonged exposure to an HFD may result in hepatic lipid accumulation and a deterioration of liver function. Obesity elevates the risk of non-alcoholic fatty liver disease and associated conditions, which are characterised by enlarged lipid droplets and decreased interstitial spaces, which compress nearby cells (19). Analysis of the liver histopathological morphology using H&E staining indicates that adipose tissue stores excess energy by increasing the size and number of adipocytes (20). In the current study, *L. reuteri* HM108 inhibited HFD-driven adipocyte growth (Figure 1F).

### 3.2 *Limosilactobacillus reuteri* HM108 attenuates serum lipid content in HFD-fed rats

**Figure 2:**
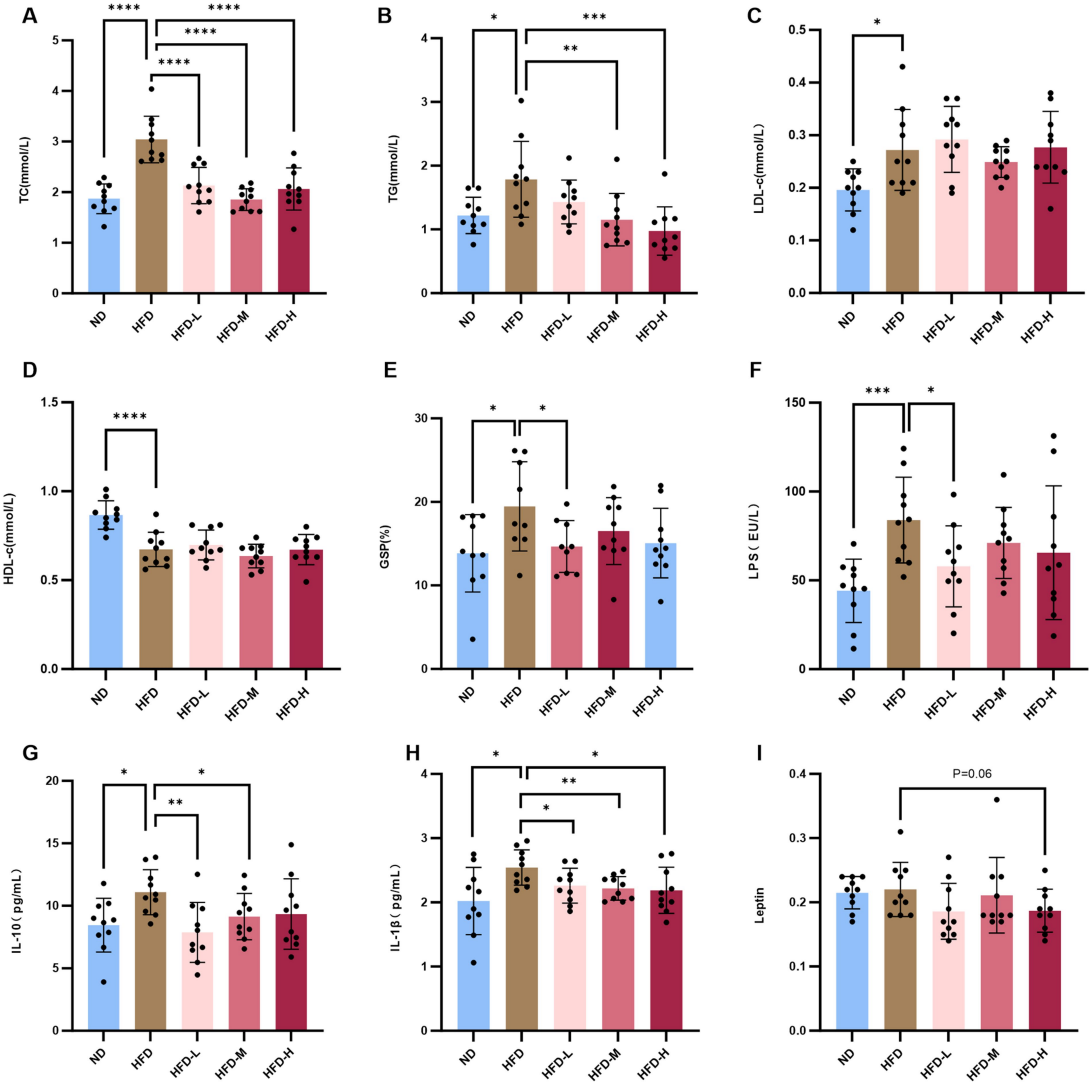
*L. reuteri* HM108 reduces serum lipid levels and inflammatory factors in HFD-fed rats. **(A)** Serum total cholesterol (TC) concentration. **(B)** Serum triglyceride (TG) concentration. **(C)** Serum low-density lipoprotein cholesterol (LDL-C) concentration. **(D)** Serum high-density lipoprotein cholesterol (HDL-C) concentration. **(E)** Serum glycated serum protein (GSP) concentration. **(F)** Serum lipopolysaccharide (LPS) concentration. **(G)** Serum interleukin-10 (IL-10) concentration. **(H)** Serum interleukin-1β (IL-1β) concentration. **(I)** Serum leptin concentration. Data are presented as mean ± SEM and analysed using unpaired *t*-tests. *p < 0.05, **p < 0.01, ***p < 0.001, and ****p < 0.0001. ND, normal diet; HFD, high-fat diet; HFD-L, high-fat + low-dose (2.5 × 10^8^ CFU/rat); HFD-M, high-fat + medium-dose (5 × 10^8^ CFU/rat); HFD-H, high-fat + high-dose (1.5 × 10^9^ CFU/rat).

Changes in lipid content in rats are shown in Figures 2A–D. After 6 w of intervention, glucose content in the HFD group was similar to that in the other groups (Supplementary Figure 1F). However, TC, TG, and LDL-C levels were higher, and HDL-C levels were lower than those in the control and ND groups (*p* < 0.05). TG and TC levels were significantly reduced in the intervention group, indicating that *L. reuteri* HM108 can improve blood lipid levels.

GSP reflects mean glycemia over the preceding 2–3 w and serves as a reliable indicator for short-term blood glucose monitoring. The GSP content in HFD rats was elevated compared to both the ND and the intervention groups with *L. reuteri* HM108, particularly the HFD-L group (Figure 2E).

These results indicate that *L. reuteri* HM108 alleviats obesity by reducing lipid and decreasing GSP contents in the blood of rats fed an HFD.

### 3.3 *Limosilactobacillus reuteri* HM108 reduces inflammatory factors in HFD-fed rats

HFD induces inflammation and dysregulation of adipocytokines as adipose tissue releases adipokines and biologically active molecules such as leptin, which affect systemic homeostasis. Most adipokines promote inflammation (21). Serum leptin, LPS, IL-6, IL-10, IL-1β, and TNF-α were quantified using ELISA (Supplementary Figures 1G,H). The findings indicated no marked alteration in TNF-α serum levels, whereas LPS, IL-6, IL-10, and IL-1β contents increased substantially in response to HFD. With *L. reuteri* HM108 treatment, the contents of LPS, IL-10, and IL-1β in HFD-induced rats were markedly reduced and restored to the contents of the ND group (Figures 2F–H). Leptin, an inflammatory factor that regulates body intake and energy expenditure, tended to decline (*p* = 0.06; Figure 2I).

The results suggest that *L. reuteri* HM108 can reduce chronic inflammation linked to obesity by decreasing serum inflammatory factor contents in a dose-dependent manner.

### 3.4 Effect of *Limosilactobacillus reuteri* HM108 on gut microbiota

**Figure 3:**
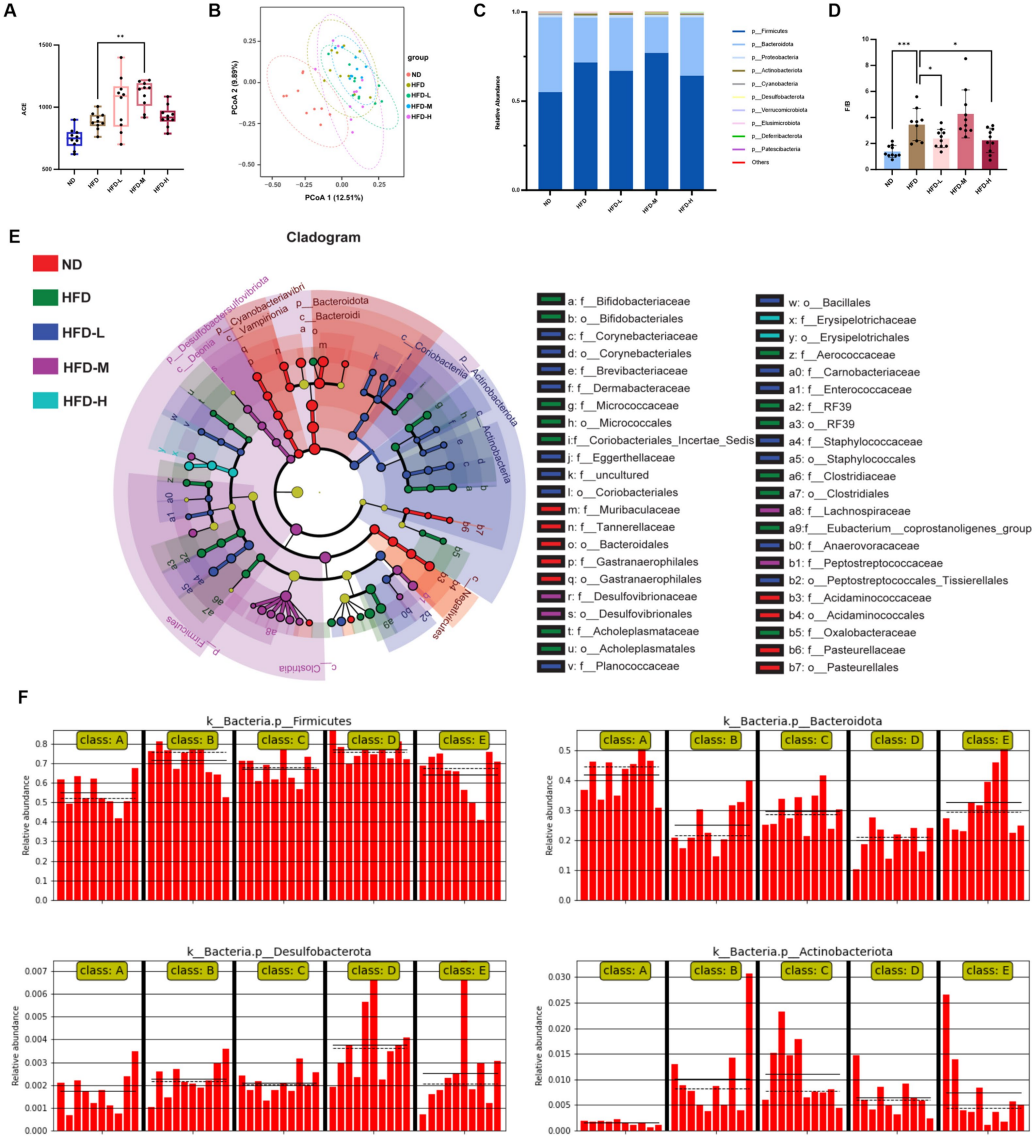
Effect of *L. reuteri* HM108 on gut microbiota in rats analysed using 16S rRNA sequencing. **(A)** ACE index in α diversity analysis of gut microbiota. **(B)** UniFrac PCoA analysis in β diversity analysis of gut microbiota based on ASV data from five groups. **(C)** Relative abundance of the intestinal flora at the phylum level. **(D)** Firmicutes/Bacteroidetes (F/B) ratio in the five groups. **(E)** LEfSe evolutionary branching maps of enteric flora in the five groups. **(F)** Relative abundance of *Firmicutes*, *Bacteroidetes*, *Desulfobacterota* and *Actinobacteriota* in LEfSe analyses. *p < 0.05, **p < 0.01, ***p < 0.001. ND, normal diet; HFD, high-fat diet; HFD-L, high-fat + low-dose (2.5 × 10^8^ CFU/rat); HFD-M, high-fat + medium-dose (5 × 10^8^ CFU/rat); HFD-H, high-fat + high-dose (1.5 × 10^9^ CFU/rat).

To determine whether *L. reuteri* HM108 affected the gut microbiota and reduced obesity, 16S rRNA sequencing was used to investigate gut microbiota composition. The α diversity ACE index indicated that *L. reuteri HM108* affected the diversity of intestinal microflora (Figure 3A), whereas the PCA of β diversity demonstrated no marked variation in gut microbiota across all HFD groups (Figure 3B). The relative abundance of the predominant intestinal flora was evaluated at the phylum level. All faecal samples were found to have a similar community structure; Firmicutes and Bacteroidetes were dominant, representing over 90% of the relative abundance. The F/B ratio rose to 3.46 ± 1.23 in obese rats on the HFD versus the ND group but dropped to 2.25 ± 0.94 in the intervention group with *L. reuteri* HM108 (Figures 3C,D).

LEfSe analysis was used to identify the microbiota altered by *L. reuteri* HM108 in HFD rats (Figures 3E,F). *Limosilactobacillus reuteri* HM108 increased the abundance of *Bacteroides*, *Prevotellaceae*, *Eubacterium*, Anaerovoracaceae, *Holdemanella*, *Enterococcaceae*, *Jeotgalicoccus*, and *Ruminococcaceae* while reducing the abundance of harmful bacteria such as *Lachnospiraceae*, *Clostridia_UCG-014*, *Butyricimonas*, *Staphylococcaceae*, and *Rothia*. The abundance of *Lactobacillus* showed no marked variation across the groups.

Functional changes in the gut microbiota with *L. reuteri* HM108 intervention were predicted using the KEGG database (Supplementary Figure 2I). Compared to the ND group, HFD consumption enriched the metabolism-related KEGG pathways, including the pentose phosphate pathway, ABC transporters, pyruvate metabolism, TCA cycle, PPAR signalling pathway, LPS biosynthesis, carbohydrate digestion, and absorption, all of which were reversed by *L. reuteri* HM108. Moreover, *L. reuteri* HM108 substantially enhanced the synthesis of several small molecules and metabolic pathways, including amino acid and polyamine metabolism; these small-molecule metabolites act as growth signals and nutrients for microbes and the host gut epithelium (22).

These results suggest that *L. reuteri* HM108 can alter the gut flora composition of HFD rats to promote the expansion of bacteria that are beneficial in the context of obesity.

### 3.5 Effects of *Limosilactobacillus reuteri* HM108 on SCFAs

SCFAs are key regulators of lipid metabolism. Propionic, acetic, and butyric acids account for >95% of intestinal SCFAs (23). GC-MS analysis of rat faeces indicated that the HFD group reduced most SCFAs (*p* < 0.05), except for butyric acid (*p* = 0.15), compared to the ND group, suggesting that HFD inhibited SCFAs production. *Limosilactobacillus reuteri* HM108 tended to increase butyric acid contents in rat faeces (*p* = 0.097) (Supplementary Figures 2A–H). These results suggest that HFD inhibits SCFAs production, which can be reversed by *L. reuteri* HM108.

### 3.6 Effects of *Limosilactobacillus reuteri* HM108 on faecal metabolites

**Figure 4:**
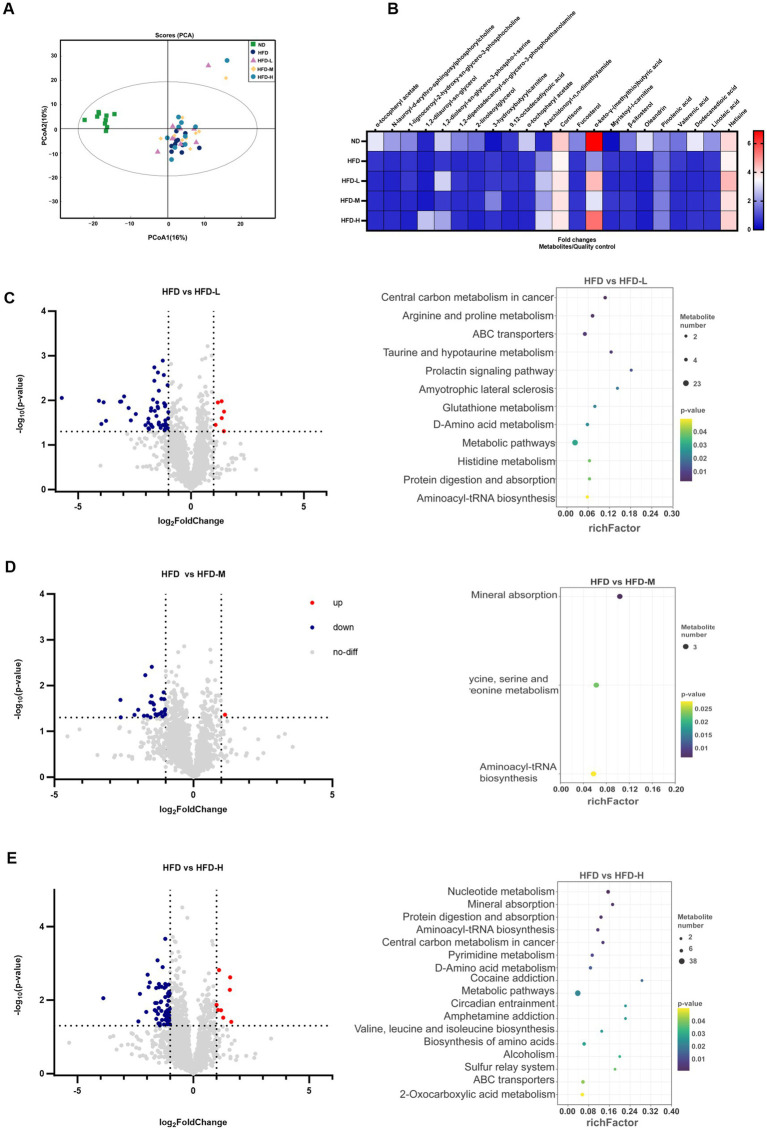
Effects of *L. reuteri* HM108 on faecal metabolites in HFD-fed rats. **(A)** Principal component analysis (PCA). **(B)** Heatmap of significant changes in faecal metabolites. **(C)** Differential metabolites and KEGG pathway prediction for HFD and HFD-L groups. **(D)** Differential metabolites and KEGG pathway prediction for HFD and HFD-M groups. **(E)** Differential metabolites and KEGG pathway prediction for HFD and HFD-H groups. ND, normal diet; HFD, high-fat diet; HFD-L, high-fat + low-dose (2.5 × 10^8^ CFU/rat); HFD-M, high-fat + medium-dose (5 × 10^8^ CFU/rat); HFD-H, high-fat + high-dose (1.5 × 10^9^ CFU/rat).

To assess the metabolic alterations caused by *L. reuteri* HM108 in HFD-induced rats, faecal samples were subjected to untargeted metabolomic profiling using UHPLC-Q-TOF MS. After combining the positive and negative ions, PCA indicated that the HFD diet altered metabolites and was screened for differential metabolites (|log_2_FC| >1 and *p* < 0.05) (Figure 4A). Compared to the HFD, 61 differential metabolites were present in the HFD-L group, 27 in the HFD-M group, and 73 in the HFD-H group. Lipids and lipid molecules with substantial changes in abundance across the five groups, including sn-glycero-3-phosphocholine, butyric acid, hetisine, and linoleic acid, were analysed using heat maps (Figure 4B).

PICRUSt2 was used to forecast the gut microbiota’s KEGG function before and after the *L. reuteri* HM108 intervention (Figures 4C–E). Relative to the HFD group, the HFD-L group exhibited 11 pathways associated with obesity: arginine and proline metabolism, ABC transporters, taurine and hypotaurine metabolism, metabolic pathways, glutathione metabolism, d-amino acid metabolism, histidine metabolism, protein digestion and absorption; and aminoacyl-tRNA biosynthesis. The HFD-M group also changed mineral absorption and glycine, serine, and threonine metabolism. The HFD-H group increased nucleotide and pyrimidine metabolism and valine, leucine, and isoleucine biosynthesis.

These results suggest that *L. reuteri* HM108 can alter the metabolites in faeces and affect pathways associated with obesity, such as energy metabolism, protein synthesis, and amino acids, thereby alleviating obesity in rats.

### 3.7 Effects of *Limosilactobacillus reuteri* HM108 on the liver transcriptome

**Figure 5:**
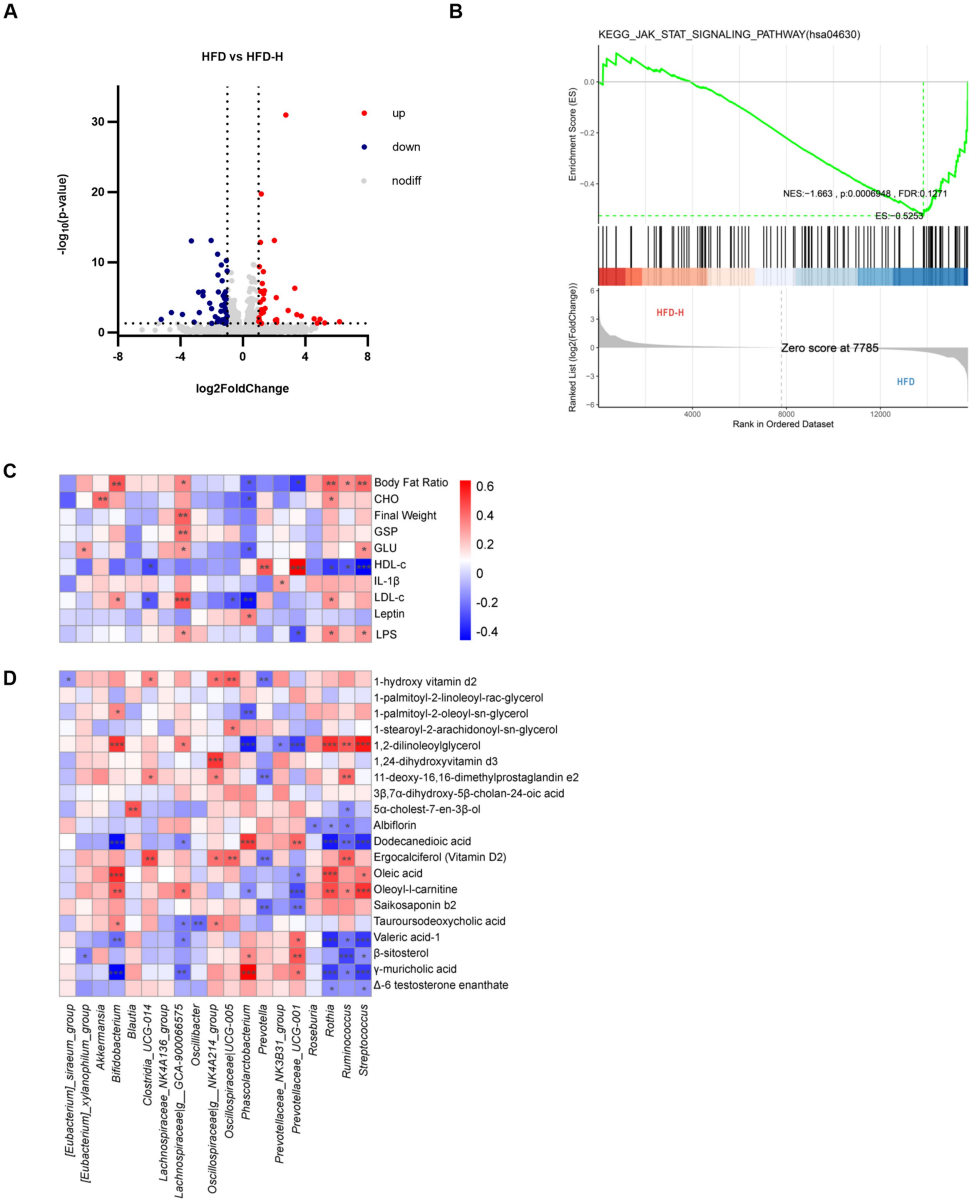
Effects of *L. reuteri* HM108 on liver transcription in HFD-fed rats and correlation analysis of gut microbiota with phenotype and metabolome. **(A)** Volcano plot of differentially expressed genes in HFD and HFD-H groups. **(B)** GSEA analysis between HFD and HFD-H groups. **(C)** Spearman correlation analysis of phenotypes with gut microbiota. **(D)** Spearman correlation analysis between gut microbiota and differential metabolites. *p < 0.05, **p < 0.01, and ***p < 0.001. HFD, high-fat diet; HFD-H, high-fat + high-dose (1.5 × 10^9^ CFU/rat).

After identifying the obesity-reducing phenotype of *L. reuteri* HM108, transcriptome analysis was conducted on liver tissues from HFD and HFD-H rats to elucidate the association between changes in gene expression related to liver tissue and obesity resulting from the HFD. A total of 81 differentially expressed genes were identified, with 33 upregulated and 48 downregulated genes. The upregulated genes included *Map3k5*, *Reg3b*, *G6pc*, *Ndst2*, *Flrt1*, *Reg3g*, *Plcd3*, and *Ndufa1*; downregulated genes included *Rgs3*, *Dact1*, *Hmgcr*, *Socs3*, *Cyp7a1*, *Foxo3*, *Lpin1*, *Cish*, *Gck*, and *Insig1* (Figure 5A). KEGG pathway enrichment analysis results are shown in Supplementary Table S1.

GSEA was examined using actual overall trends rather than specific differential gene thresholds. In this research, compared to the HFD group, the expression of JAK-STAT signalling pathway in liver tissue decreased in the intervention group (Figure 5B). The main genes involved in this pathway were *Myc* (ENSRNOG00000004500), *Cish* (ENSRNOG00000029543), and *Osmr* (ENSRNOG00000033192).

In this study, compared to the HFD group, the expression of JAK-STAT signalling pathway in liver tissue decreased in the intervention group of *Lactobacillus rhamnosus* HM108.

The results indicate that intervention with *L. reuteri* HM108 can effectively inhibit the JAK-STAT signalling pathway, with the important downregulation of *Myc*, *Cish*, and *Osmr* genes.

### 3.8 Associations between phenotype, microbiome, and metabolome

Obesity and metabolic disorders induced by an HFD largely stem from a gut microbiota imbalance. To study the effect of *L. reuteri* HM108 on obesity via the gut microbiota, 20 gut microbiota with marked changes in genus-level abundance before and after the intervention were selected for Spearman’s correlation analysis (Figure 5C). Although Lachnospiraceae are SCFAs producers (24), they are more abundant in obese and diabetic mice and humans (25). The correlation analysis indicated that *Lachnospiraceae_GCA-900066575* exhibited a positive association with several markers, including body fat ratio, body weight, GSP, glucose, low-density lipoprotein, and LPS. This suggests that it plays a key role in the development of obesity.

*Limosilactobacillus reuteri* HM108 affected the composition of gut microbes and metabolite profiles. Therefore, Spearman’s correlation analysis was conducted for these 20 gut microorganisms and 20 significantly different metabolites associated with obesity (Figure 5D). The important intestinal bacterial genus, *Prevotellaceae_UCG-001* was positively correlated with valeric acid-1, β-sitosterol, and γ-muricholic acid and negatively correlated with oleic acid, oleoyl-l-carnitine, and saikosaponin b2. *Prevotella* was correlated with 1-hydroxyvitamin D, 11-deoxy-16,16-dimethylprostaglandin e2, ergocalciferol (vitamin D2), and saikosaponin b2.

## 4 Discussion

Globally, obesity presents a major health crisis and is increasingly regarded as a non-communicable pandemic (26). Obesity exacerbates cardiovascular disease progression and mortality beyond its association with risk factors such as dyslipidaemia, type 2 diabetes, and hypertension (27). However, behavioural and pharmacological therapies yield only 3–10% body weight loss, and maintaining this reduction is challenging (28). Numerous studies have shown that probiotic strains, either alone or in combination with other compounds, can exert anti-obesity effects by regulating gut microbiota, enhancing insulin sensitivity, and modulating appetite (29).

A calorie-dense, fatty, and sugary diet with low fibre content is a primary driver of weight gain. The rodent obesity model closely reflects human physiology and weight regulation (30). In the current study, a high-sugar and HFD containing 15% sucrose and 15% lard was used to induce obesity in male SPF rats and investigate the ameliorative effects of different doses of *L. reuteri* HM108 via gavage. The results showed that *L. reuteri* HM108 reduced body weight gain, inhibited adipocyte enlargement, and regulated blood lipid content in a dose-dependent manner.

An HFD induces chronic inflammation in obese individuals (31). Obesity disrupts gut microbiota balance, increases intestinal permeability, and facilitates the translocation of metabolic waste and microbial toxins into the bloodstream. This process stimulates cytokine release, contributing to chronic inflammation and insulin resistance (32). In the current study, HFD-induced obesity in rats was accompanied by the upregulation of inflammatory factors, whereas *L. reuteri* HM108 substantially reduced serum LPS and IL-1β levels.

HFD-induced obesity and associated metabolic disorders are characterised by gut flora dysbiosis, which affects nutrition, digestion, and energy metabolism (33). The primary gastrointestinal bacterial phyla were Firmicutes and Bacteroidetes; the F/B ratio serves as a key biomarker of malnutrition and metabolism-related disorders. In the current study, HFD-induced gut flora imbalance was manifested by an increase in Firmicutes, a decrease in Bacteroidetes, and a marked elevation in the F/B ratio, consistent with previous findings (34). *Limosilactobacillus reuteri* HM108 increased the prevalence of *Bacteroidetes* while reducing *Firmicutes*, leading to a marked decrease in the F/B ratio in HFD-fed rats. This shift substantially altered gut microbiota composition. *Limosilactobacillus reuteri* HM108 also increased the abundance of *Bacteroides*, *Prevotellaceae*, *Eubacterium*, *Anaerovoracaceae*, *g-Holdemanella*, *Enterococcaceae*, *Jeotgalicoccus*, and *Ruminococcaceae*. Previous studies have shown that *Bacteroides* (35), *Lachnospiraceae* (36), *Prevotellaceae* (37), and *Ruminiclostridium* (38) are negatively correlated with body weight, lipid metabolism, obesity, and related diseases. *Holdemanella biformis* has been shown to improve glucose tolerance in obese mice (39). *Limosilactobacillus reuteri* HM108 reduced the abundance of *Lachnospiraceae*, *Clostridia_UCG-014*, *Butyricimonas*, *Staphylococcaceae*, *Rothia*, and other harmful bacteria. *Clostridia_UCG-014* has been positively correlated with blood glucose concentration and is more abundant in diabetic and obese mice (40). The primary functions of these gut microbiota include regulating polysaccharide biodegradation, SCFAs production, specific LPS enrichment, and the synthesis of vitamins and essential amino acids. These processes influence energy uptake, hunger signals, fat accumulation, systemic inflammation, and circadian rhythms, thereby alleviating obesity (39). Therefore, *L. reuteri* HM108 positively impacts diet-induced gut microbiota dysbiosis.

Alterations in gut microbiota composition influence SCFAs levels, as most SCFAs originate from the microbial fermentation of indigestible dietary components (40). *Bacteroides*, enhanced by *L. reuteri* HM108, reduce inflammation through propionate production (41). *Lactobacillaceae*, *Ruminococcaceae*, and *Lachnospiraceae* hydrolyse polysaccharides to produce butyrate and other SCFAs (42). *Jeotgalicoccus* is positively correlated with acetate, propionate, and butyrate concentrations (43). *Eubacterium* not only produces SCFAs but also regulates bile acid metabolism to alleviate obesity (44). These SCFAs play key roles in maintaining intestinal integrity, cellular function, and immune responses, potentially influencing the development of cardiovascular and metabolic diseases associated with high-fat or high-sugar intake (45).

Gut microbiota produces SCFAs and other metabolites that act as messengers between microbes and their hosts. In this study, *L. reuteri* HM108 intervention substantially ameliorated HFD-induced lipid metabolism disorders in obese rats. The following metabolites were involved: phosphatidylcholine and phosphatidylethanolamine, which regulate hepatic insulin signalling (46); linoleic acid, which influences adipocyte and skeletal muscle metabolism (47); 7α-hydroxy-4-cholesten-3-one, lithocholic acid, and taurine, which are intermediates in cholesterol metabolism via bile acids (48); and other substances that may improve HDL cholesterol levels, such as 1-hydroxy vitamin D2, L-ascorbic acid, cedrol (49), and betaine. Additionally, *L. reuteri* HM108 modulated the amino acid metabolic pathways that influence fatty acid synthesis *in vivo* more efficiently than glucose (50).

Probiotics influence signalling pathways that regulate gene expression, impacting energy metabolism and inflammation. Transcriptome analysis indicated that *L. reuteri* HM108 strongly inhibited the JAK-STAT signalling pathway, which regulates multiple downstream biological processes, including adipogenesis, inflammation, and apoptosis (51). Aberrant activation of this pathway leads to dysregulation of hepatic gluconeogenesis, hepatic steatosis, and insulin resistance (52, 53). Mice fed an HFD exhibit markedly increased fat deposition when deficient in the *socs3* gene, whereas JAK pathway inhibition attenuates HFD-induced obesity (54). Inhibiting the JAK/STAT signalling pathway not only alleviates HFD-induced obesity but also improves the chronic inflammatory state of adipose tissue in obese individuals. Cytokines released during obesity can activate JAK 1/2, which in turn phosphorylate STAT proteins, a pathway persistently activated in various tumour cells (55). Blocking JAK-STAT signalling substantially reduces intestinal inflammation and improves barrier function in HFD-fed mice (53).

*Limosilactobacillus reuteri* HM108, especially at high doses, inhibited weight gain and reduced adipose tissue enlargement in HFD-fed rats. The findings indicate that *L. reuteri* HM108 dose-dependently reduces HFD-induced obesity-related phenotypes in rats. However, additional clinical studies are required to validate the efficacy of *L. reuteri* against HFD-induced obesity in humans, alongside a more comprehensive investigation of its underlying molecular mechanisms.

## 5 Conclusion

*Limosilactobacillus reuteri* HM108 dose-dependently attenuated body weight and adipocyte hypertrophy, reduced inflammatory factors and blood lipid content in HFD-fed rats, and alleviated HFD-induced obesity through modifications in the intestinal flora, including lowering the F/B ratio and altering metabolite profiles. The JAK-STAT signalling pathway was inhibited. The results offer a theoretical foundation and practical insights for utilising *L. reuteri* HM108 in interventions for HFD-induced obesity.

## Data Availability

The original contributions presented in the study are publicly available. This data can be found in the Mendeley Data repository: 10.17632/3jfj6d4t2g.1.

## Footnotes

^1^ https://www.genome.jp/kegg/ligand.html

## References

[1] Zhang N Wang Q Lin F Zheng B Huang Y Yang Y. Neoagarotetraose alleviates high fat diet induced obesity via white adipocytes browning and regulation of gut microbiota. Carbohydr Polym. (2022) 296:119903. doi: 10.1016/j.carbpol.2022.119903, PMID: 36087969

[2] Zhang H Chen S Yang L Zhang S Qin L Jiang H. Distinct gut microbiota and arachidonic acid metabolism in obesity-prone and obesity-resistant mice with a high-fat diet. Nutrients. (2024) 16:1579. doi: 10.3390/nu16111579, PMID: 38892512 PMC11174461

[3] Ke W Flay KJ Huang X Hu X Chen F Li C. Polysaccharides from *Platycodon grandiflorus* attenuates high-fat diet induced obesity in mice through targeting gut microbiota. Biomed Pharmacother. (2023) 166:115318. doi: 10.1016/j.biopha.2023.115318, PMID: 37572640

[4] Qin Q Shou J Li M Gu M Meng Z Xu P. Stk24 protects against obesity-associated metabolic disorders by disrupting the NLRP3 inflammasome. Cell Rep. (2021) 35:109161. doi: 10.1016/j.celrep.2021.109161, PMID: 34038725

[5] Kober A Saha S Ayyash M Namai F Nishiyama K Yoda K. Insights into the anti-adipogenic and anti-inflammatory potentialities of probiotics against obesity. Nutrients. (2024) 16:1373. doi: 10.3390/nu16091373, PMID: 38732619 PMC11085650

[6] van Zyl WF Deane SM Dicks LMT. Molecular insights into probiotic mechanisms of action employed against intestinal pathogenic bacteria. Gut Microbes. (2020) 12:1831339. doi: 10.1080/19490976.2020.1831339, PMID: 33112695 PMC7595611

[7] Chavoya-Guardado MA Vasquez-Garibay EM Ruiz-Quezada SL Ramírez-Cordero MI Larrosa-Haro A Castro-Albarran J. Firmicutes, Bacteroidetes and Actinobacteria in human milk and maternal adiposity. Nutrients. (2022) 14:2887. doi: 10.3390/nu14142887, PMID: 35889844 PMC9315738

[8] Green M Arora K Prakash S. Microbial medicine: prebiotic and probiotic functional foods to target obesity and metabolic syndrome. Int J Mol Sci. (2020) 21:2890. doi: 10.3390/ijms21082890, PMID: 32326175 PMC7215979

[9] Cai Y Liu P Zhou X Yuan J Chen Q. Probiotics therapy show significant improvement in obesity and neurobehavioral disorders symptoms. Front Cell Infect Microbiol. (2023) 13:1178399. doi: 10.3389/fcimb.2023.1178399, PMID: 37249983 PMC10213414

[10] Wei B Zhang B Du AQ Zhou ZY Lu DZ Zhu ZH. *Saccharina japonica* fucan suppresses high fat diet-induced obesity and enriches fucoidan-degrading gut bacteria. Carbohydr Polym. (2022) 290:119411. doi: 10.1016/j.carbpol.2022.119411, PMID: 35550744

[11] Cani PD. Targeting gut microbiota with a complex mix of dietary fibers improves metabolic diseases. Kidney Int. (2019) 95:14–6. doi: 10.1016/j.kint.2018.11.012, PMID: 30606413

[12] Hays KE Pfaffinger JM Ryznar R. The interplay between gut microbiota, short-chain fatty acids, and implications for host health and disease. Gut Microbes. (2024) 16:2393270. doi: 10.1080/19490976.2024.2393270, PMID: 39284033 PMC11407412

[13] Liu Y Gao Y Ma F Sun M Mu G Tuo Y. The ameliorative effect of *Lactobacillus plantarum* Y44 oral administration on inflammation and lipid metabolism in obese mice fed with a high fat diet. Food Funct. (2020) 11:5024–39. doi: 10.1039/d0fo00439a, PMID: 32530448

[14] Cerdó T García-Santos JA Bermúdez MG Campoy C. The role of probiotics and prebiotics in the prevention and treatment of obesity. Nutrients. (2019) 11:635. doi: 10.3390/nu11030635, PMID: 30875987 PMC6470608

[15] Mu Q Tavella VJ Luo XM. Role of *Lactobacillus reuteri* in human health and diseases. Front Microbiol. (2018) 9. doi: 10.3389/fmicb.2018.00757, PMID: 29725324 PMC5917019

[16] Li C Su Z Chen Z Cao J Liu X Xu F. *Lactobacillus reuteri* strain 8008 attenuated the aggravation of depressive-like behavior induced by CUMS in high-fat diet-fed mice through regulating the gut microbiota. Front Pharmacol. (2023) 14:1149185. doi: 10.3389/fphar.2023.1149185, PMID: 37050901 PMC10083334

[17] Yang B Zheng F Stanton C Ross RP Zhao J Zhang H. *Lactobacillus reuteri* Fynlj109l1 attenuating metabolic syndrome in mice via gut microbiota modulation and alleviating inflammation. Foods. (2021) 10:2081. doi: 10.3390/foods10092081, PMID: 34574191 PMC8469823

[18] Fåk F Bäckhed F. *Lactobacillus reuteri* prevents diet-induced obesity, but not atherosclerosis, in a strain dependent fashion in Apoe−/− mice. PLoS One. (2012) 7:e46837. doi: 10.1371/journal.pone.0046837, PMID: 23056479 PMC3467285

[19] Hong Y Song G Feng X Niu J Wang L Yang C. The probiotic *Kluyveromyces lactis* JSA 18 alleviates obesity and hyperlipidemia in high-fat diet C57BL/6J mice. Foods. (2024) 13:1124. doi: 10.3390/foods13071124, PMID: 38611428 PMC11011337

[20] Ji T Fang B Wu F Liu Y Cheng L Li Y. Diet change improves obesity and lipid deposition in high-fat diet-induced mice. Nutrients. (2023) 15:4978. doi: 10.3390/nu15234978, PMID: 38068835 PMC10708053

[21] Ohashi K Shibata R Murohara T Ouchi N. Role of anti-inflammatory adipokines in obesity-related diseases. Trends Endocrinol Metab. (2014) 25:348–55. doi: 10.1016/j.tem.2014.03.009, PMID: 24746980

[22] Wang P Gao J Ke W Wang J Li D Liu R. Resveratrol reduces obesity in high-fat diet-fed mice via modulating the composition and metabolic function of the gut microbiota. Free Radic Biol Med. (2020) 156:83–98. doi: 10.1016/j.freeradbiomed.2020.04.013, PMID: 32305646

[23] Cong J Zhou P Zhang R. Intestinal microbiota-derived short chain fatty acids in host health and disease. Nutrients. (2022) 14:1977. doi: 10.3390/nu14091977, PMID: 35565943 PMC9105144

[24] Vacca M Celano G Calabrese FM Portincasa P Gobbetti M De Angelis M. The controversial role of human gut Lachnospiraceae. Microorganisms. (2020) 8:573. doi: 10.3390/microorganisms8040573, PMID: 32326636 PMC7232163

[25] Takeuchi T Kameyama K Miyauchi E Nakanishi Y Kanaya T Fujii T. Fatty acid overproduction by gut commensal microbiota exacerbates obesity. Cell Metab. (2023) 35:361–75.e9. doi: 10.1016/j.cmet.2022.12.013, PMID: 36652945

[26] Blüher M. Obesity: global epidemiology and pathogenesis. Nat Rev Endocrinol. (2019) 15:288–98. doi: 10.1038/s41574-019-0176-8, PMID: 30814686

[27] Piché ME Tchernof A Després JP. Obesity phenotypes, diabetes, and cardiovascular diseases. Circ Res. (2020) 126:1477–500. doi: 10.1161/circresaha.120.316101, PMID: 32437302

[28] Blüher M. Metabolically healthy obesity. Endocr Rev. (2020) 41:3. doi: 10.1210/endrev/bnaa004, PMID: 32128581 PMC7098708

[29] Abenavoli L Scarpellini E Colica C Boccuto L Salehi B Sharifi-Rad J. Gut microbiota and obesity: a role for probiotics. Nutrients. (2019) 11:2690. doi: 10.3390/nu11112690, PMID: 31703257 PMC6893459

[30] Speakman JR. Use of high-fat diets to study rodent obesity as a model of human obesity. Int J Obes. (2019) 43:1491–2. doi: 10.1038/s41366-019-0363-7, PMID: 30967607

[31] Olofsson LE Bäckhed F. The metabolic role and therapeutic potential of the microbiome. Endocr Rev. (2022) 43:907–26. doi: 10.1210/endrev/bnac004, PMID: 35094076 PMC9512151

[32] Nakanishi T Fukui H Wang X Nishiumi S Yokota H Makizaki Y. Effect of a high-fat diet on the small-intestinal environment and mucosal integrity in the gut-liver axis. Cells. (2021) 10:3168. doi: 10.3390/cells10113168, PMID: 34831391 PMC8622719

[33] Amabebe E Robert FO Agbalalah T Orubu ESF. Microbial dysbiosis-induced obesity: role of gut microbiota in homoeostasis of energy metabolism. Br J Nutr. (2020) 123:1127–37. doi: 10.1017/s0007114520000380, PMID: 32008579

[34] Stojanov S Berlec A Štrukelj B. The influence of probiotics on the Firmicutes/Bacteroidetes ratio in the treatment of obesity and inflammatory bowel disease. Microorganisms. (2020) 8:1715. doi: 10.3390/microorganisms8111715, PMID: 33139627 PMC7692443

[35] Li H Wang XK Tang M Lei L Li JR Sun H. *Bacteroides thetaiotaomicron* ameliorates mouse hepatic steatosis through regulating gut microbial composition, gut-liver folate and unsaturated fatty acids metabolism. Gut Microbes. (2024) 16:2304159. doi: 10.1080/19490976.2024.2304159, PMID: 38277137 PMC10824146

[36] Xiang X Zhou X Wang W Zhou Y Zhou X Deng S. Effect of Antarctic krill phospholipid (Kopl) on high fat diet-induced obesity in mice. Food Res Int. (2021) 148:110456. doi: 10.1016/j.foodres.2021.110456, PMID: 34507719

[37] Li Q Liu W Feng Y Hou H Zhang Z Yu Q. Radix *Puerariae thomsonii* polysaccharide (RPP) improves inflammation and lipid peroxidation in alcohol and high-fat diet mice by regulating gut microbiota. Int J Biol Macromol. (2022) 209:858–70. doi: 10.1016/j.ijbiomac.2022.04.067, PMID: 35439478

[38] Liu F Tang X Mao B Zhang Q Zhao J Cui S. Ethanol extract of licorice alleviates HFD-induced liver fat accumulation in association with modulation of gut microbiota and intestinal metabolites in obesity mice. Nutrients. (2022) 14:4180. doi: 10.3390/nu14194180, PMID: 36235833 PMC9572531

[39] Liu BN Liu XT Liang ZH Wang JH. Gut microbiota in obesity. World J Gastroenterol. (2021) 27:3837–50. doi: 10.3748/wjg.v27.i25.3837, PMID: 34321848 PMC8291023

[40] Canfora EE Jocken JW Blaak EE. Short-chain fatty acids in control of body weight and insulin sensitivity. Nat Rev Endocrinol. (2015) 11:577–91. doi: 10.1038/nrendo.2015.128, PMID: 26260141

[41] Price CE Valls RA Ramsey AR Loeven NA Jones JT Barrack KE. Intestinal bacteroides modulates inflammation, systemic cytokines, and microbial ecology via propionate in a mouse model of cystic fibrosis. mBio. (2024) 15:e0314423. doi: 10.1128/mbio.03144-23 38179971 PMC10865972

[42] Fusco W Lorenzo MB Cintoni M Porcari S Rinninella E Kaitsas F. Short-chain fatty-acid-producing bacteria: key components of the human gut microbiota. Nutrients. (2023) 15:2211. doi: 10.3390/nu15092211, PMID: 37432351 PMC10180739

[43] Pei L Liu W Liu L Wang X Jiang L Chen Z. Morel (*Morchella* spp.) intake alters gut microbial community and short-chain fatty acid profiles in mice. Front Nutr. (2023) 10:1237237. doi: 10.3389/fnut.2023.1237237, PMID: 37810928 PMC10556497

[44] Mukherjee A Lordan C Ross RP Cotter PD. Gut microbes from the phylogenetically diverse genus *Eubacterium* and their various contributions to gut health. Gut Microbes. (2020) 12:1802866. doi: 10.1080/19490976.2020.1802866, PMID: 32835590 PMC7524325

[45] Kaye DM Shihata WA Jama HA Tsyganov K Ziemann M Kiriazis H. Deficiency of prebiotic fiber and insufficient signaling through gut metabolite-sensing receptors leads to cardiovascular disease. Circulation. (2020) 141:1393–403. doi: 10.1161/circulationaha.119.043081, PMID: 32093510

[46] van der Veen JN Lingrell S McCloskey N LeBlond ND Galleguillos D Zhao YY. A role for phosphatidylcholine and phosphatidylethanolamine in hepatic insulin signaling. FASEB J. (2019) 33:5045–57. doi: 10.1096/fj.201802117R, PMID: 30615497

[47] Basak S Duttaroy AK. Conjugated linoleic acid and its beneficial effects in obesity, cardiovascular disease, and cancer. Nutrients. (2020) 12:1913. doi: 10.3390/nu12071913, PMID: 32605287 PMC7401241

[48] Wei W Lyu X Markhard AL Fu S Mardjuki RE Cavanagh PE. PTER is a N-acetyltaurine hydrolase that regulates feeding and obesity. Nature. (2024) 633:182–8. doi: 10.1038/s41586-024-07801-6, PMID: 39112712 PMC11374699

[49] Zhao Y Li M Guo J Fang J Geng R Wang Y. Cedrol, a major component of cedarwood oil, ameliorates high-fat diet-induced obesity in mice. Mol Nutr Food Res. (2023) 67:e2200665. doi: 10.1002/mnfr.202200665, PMID: 37143286

[50] Liao Y Chen Q Liu L Huang H Sun J Bai X. Amino acid is a major carbon source for hepatic lipogenesis. Cell Metab. (2024) 36:2437–48.e8. doi: 10.1016/j.cmet.2024.10.001, PMID: 39461344

[51] Hu X Li J Fu M Zhao X Wang W. The JAK/STAT signaling pathway: from bench to clinic. Signal Transduct Target Ther. (2021) 6:402. doi: 10.1038/s41392-021-00791-1, PMID: 34824210 PMC8617206

[52] Dodington DW Desai HR Woo M. JAK/STAT—emerging players in metabolism. Trends Endocrinol Metab. (2018) 29:55–65. doi: 10.1016/j.tem.2017.11.001, PMID: 29191719

[53] Zong X Zhang H Zhu L Deehan EC Fu J Wang Y. *Auricularia auricula* polysaccharides attenuate obesity in mice through gut commensal *Papillibacter cinnamivorans*. J Adv Res. (2023) 52:203–18. doi: 10.1016/j.jare.2023.08.003, PMID: 37549868 PMC10555930

[54] Liu S Jiang W Liu C Guo S Wang H Chang X. Chinese chestnut shell polyphenol extract regulates the JAK2/STAT3 pathway to alleviate high-fat diet-induced, leptin-resistant obesity in mice. Food Funct. (2023) 14:4807–23. doi: 10.1039/d3fo00604b, PMID: 37128963

[55] Yang M Wu S Cai W Ming X Zhou Y Chen X. Hypoxia-induced MIF induces dysregulation of lipid metabolism in Hep2 laryngocarcinoma through the IL-6/JAK-STAT pathway. Lipids Health Dis. (2022) 21:82. doi: 10.1186/s12944-022-01693-z, PMID: 36042480 PMC9426221

