# Supplementary material for: Limosilactobacillus reuteri HM108 alleviates obesity in rats fed a high-fat diet by modulating the gut microbiota, metabolites, and inhibiting the JAK-STAT signalling pathway
Source: Front Nutr. 2025 Jun 25;12:1597334. doi: 10.3389/fnut.2025.1597334 (PMC12238095; doi:10.3389/fnut.2025.1597334)
Supplement: Supplementary file 1 [file Supplementary_file_1.docx]

Supplementary Material

# Supplementary Figures and Tables

## Supplementary Figures


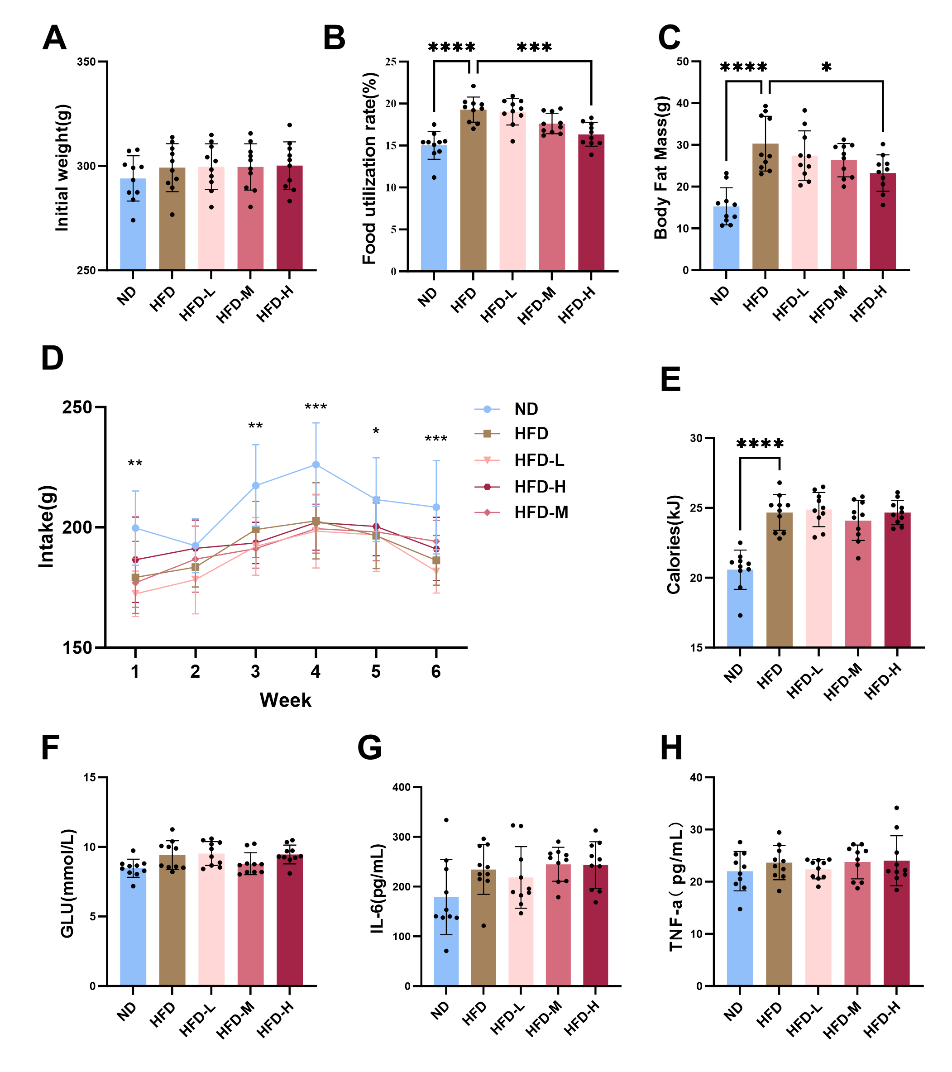


**Supplementary Figure 1.** *L. reuteri* HM108 ameliorates HFD-induced obesity in rats. (A) Initial body weight. (B) Food utilisation. (C) Body fat weight. (D) Food intake. (E) Total caloric intake. (F) Blood glucose. (G) IL-6. (H) TNF-α. Data are presented as mean ± SEM. Line graphs were analysed using two-way ANOVA, and histograms were analysed using unpaired t-tests. * Significant difference between HFD and HFD-M groups; * p <0.05,*** p <0.001, **** p <0.0001.


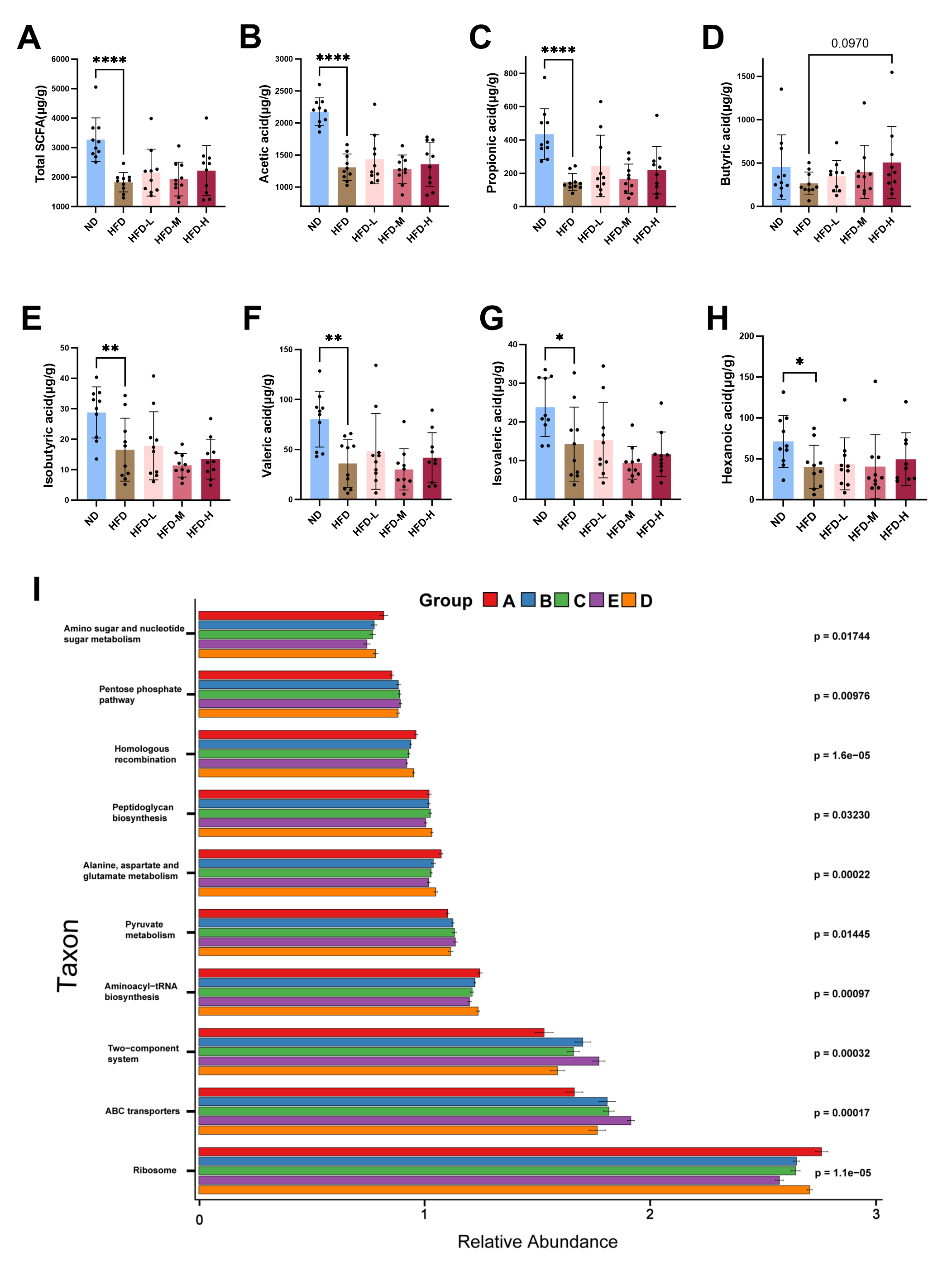


**Supplementary Figure 2.** *L. reuteri* HM108 improves HFD-induced short-chain fatty acid levels and gut microbiota function. (A) Total SCFA. (B) Acetic acid. (C) Propionic acid. (D) Butyric acid. (E) Isobutyric acid. (F) Valeric acid. (G) Isovaleric acid. (H) Hexanoic acid. (I) KEGG pathway prediction of gut microbiota. Data are presented mean ± SEM. Histograms were analysed using unpaired t-tests.* p <0.05,*** p <0.001,**** p <0.0001.

## Supplementary Tables

Table S1: annotation of DEGs in liver to the corresponding signaling pathways based on KEGG pathway enrichment analysis (p<0.05). Genes involved in 2 or more KEGG pathways are in bold (n=5).

| Pathway | Pvalue | Count | Genes |
| --- | --- | --- | --- |
| Maturity onset diabetes of the young | 0.00017916 | 3 | **Gck, Foxa2, Hhex** |
| Endometrial cancer | 0.00036766 | 4 | **Foxo3, Myc, Gadd45g, Gadd45a** |
| Prolactin signaling pathway | 0.00044642 | 4 | **Foxo3, Gck, Socs3, Cish** |
| Breast cancer | 0.00076006 | 6 | **Myc, Gadd45g, Fzd5, Gadd45a,** Dll1**, Hhex** |
| Transcriptional misregulation in cancer | 0.00108713 | 5 | **Myc,** Defa9**, Gadd45g, Dusp6, Gadd45a** |
| Thyroid cancer | 0.00144168 | 3 | **Myc, Gadd45g, Gadd45a** |
| Basal cell carcinoma | 0.00396781 | 4 | **Gadd45g, Fzd5, Gadd45a, Map3k5** |
| MAPK signaling pathway | 0.00511149 | 5 | **Myc, Gadd45g, Epha2, Dusp6, Gadd45a** |
| FoxO signaling pathway | 0.0056162 | 4 | **Foxo3, G6pc, Gadd45g, Gadd45a** |
| Central carbon metabolism in cancer | 0.00696695 | 3 | **Myc, Gck,** Slc7a5 |
| Gastric cancer | 0.00722526 | 4 | **Myc, Gadd45g, Fzd5, Gadd45a** |
| JAK-STAT signaling pathway | 0.00742099 | 4 | **Myc, Socs3, Cish, Osmr** |
| Non-small cell lung cancer | 0.00852469 | 4 | **Foxo3, Gadd45g, Gadd45a, Osmr** |
| PI3K-Akt signaling pathway | 0.00897651 | 5 | **Foxo3, Myc, G6pc,** Lpar6**, Epha2** |
| PPAR signaling pathway | 0.00955003 | 3 | Ubc, Me3**, Cyp7a1** |
| Chronic myeloid leukemia | 0.01027202 | 3 | **Myc, Gadd45g, Gadd45a** |
| Starch and sucrose metabolism | 0.01110875 | 2 | **G6pc, Gck** |
| Galactose metabolism | 0.01191838 | 2 | **G6pc, Gck** |
| Colorectal cancer | 0.01347288 | 3 | **Myc, Gadd45g, Gadd45a** |
| Bile secretion | 0.01620779 | 3 | **Hmgcr,** Nr0b2**, Cyp7a1** |
| Small cell lung cancer | 0.01620779 | 3 | **Myc, Gadd45g, Gadd45a** |
| Circadian rhythm | 0.01633828 | 2 | Bhlhe40, Nr1d1 |
| Hepatocellular carcinoma | 0.01731418 | 4 | **Myc, Gadd45g, Fzd5, Gadd45a** |
| Cellular senescence | 0.01765845 | 4 | **Foxo3, Myc, Gadd45g, Gadd45a** |
| Insulin resistance | 0.021414 | 3 | **G6pc, Socs3,** Trib3 |
| Neomycin, kanamycin and gentamicin biosynthesis | 0.02367442 | 1 | **Gck** |
| Type II diabetes mellitus | 0.02691543 | 2 | **Gck, Socs3** |
| Signaling pathways regulating pluripotency of stem cells | 0.03007586 | 3 | **Myc,** Tbx3**, Fzd5** |
| AMPK signaling pathway | 0.03214026 | 3 | **Foxo3, G6pc, Hmgcr** |
| Alcoholic liver disease | 0.03574318 | 3 | **Foxo3,** Lpin1**, Map3k5** |
| Insulin signaling pathway | 0.03800175 | 3 | **G6pc, Gck, Socs3** |
| Apoptosis | 0.04192607 | 3 | **Gadd45g, Gadd45a, Map3k5** |
| Glycolysis / Gluconeogenesis | 0.0480489 | 2 | **G6pc, Gck** |
| Longevity regulating pathway - multiple species | 0.0480489 | 2 | **Foxo3, Foxa2** |
